# Cytokeratin positivity in myxopapillary ependymoma – a potential diagnostic pitfall

**DOI:** 10.1186/1746-1596-3-40

**Published:** 2008-10-19

**Authors:** Sundus A Hussein, Monalisa Sur

**Affiliations:** 1 Department of Pathology and Molecular Medicine, McMaster University, Hamilton, Ontario, Canada; 2 Department of Anatomical Pathology, Henderson General Hospital, Hamilton, Ontario, Canada

## Abstract

**Background:**

Myxopapillary ependymomas (MPE) occur in the filum terminale of the spinal cord, but also present in extra-spinal locations such as subcutaneous tissue and brain. They are slow growing grade I gliomas. Areas of solid growth pattern with aggregates of cells with "epithelioid morphology" seen in MPE can mimic metastatic carcinoma. The presence of occasional cells with clear cytoplasm and morphology can resemble Chordoma. Diagnosis can be missed due to these morphological similarities, which could affect patient management and hence, long term survival.

**Case presentation:**

We describe two cases of MPE with cytokeratin (AE1 AE3, CAM 5.2, Cytokeratin 7 and cytokeratin 20) expression.

**Conclusion:**

MPE can be positive for Cytokeratins (CAM 5.2, AE1 AE3, CK7) and focally for EMA, which could be misdiagnosed as metastatic carcinoma. In cases demonstrating epithelioid and clear cell morphology, the diagnosis of MPE should be made in conjunction with histology, proper immunohistochemical profile which includes co-expression of GFAP, S-100 protein and epithelial markers, radiologic findings and site. It is important to be aware of the cytokeratin profile in MPE to avoid erroneous diagnosis with other tumour entities.

## Background

Myxopapillary ependymomas (MPE) generally occur in the filum terminale of the spinal cord, however, they have been described in extra-spinal locations such as subcutaneous tissue [1] and brain [2]. They are slow growing gliomas corresponding to WHO grade I [3]. The classical morphology shows papillae embedded in a myxoid/mucoid background. Each papilla contains a central fibrovascular core and is lined by cuboidal to elongated cells, occasionally showing clear cytoplasm. Microcysts are also present. The myxoid background contains both neutral and acidic mucopolysaccharides. Prognosis depends on the completeness of excision [4]. However, areas of solid growth pattern with aggregates of cells with "epithelioid morphology" can also be encountered which can mimic metastatic carcinoma. In addition, the presence of cells with clear cytoplasm can also be mistaken for chordoma. Diagnosis is easily missed due to these morphological similarities, which could affect patient management and hence, long term survival. A good number of studies have reported the immunophenotype of MPE and differential diagnosis of MPE aided by immunohistochemical stains [5-7]. Several studies have reported absence of cytokeratin expression in MPE [8-10].

## Case presentation

We describe two cases of MPE with cytokeratin expression. Two female patients, aged 46 and 72 years respectively presented with low back pain. Magnetic resonance imaging (MRI) of the spine indicated the presence of a large intra spinal mass in the central canal extending from L3–L5 in the former patient and a tumour attached to filum terminale in the latter. Both underwent neurosurgical removal of the tumor.

## Pathological findings

**Figure 1:**
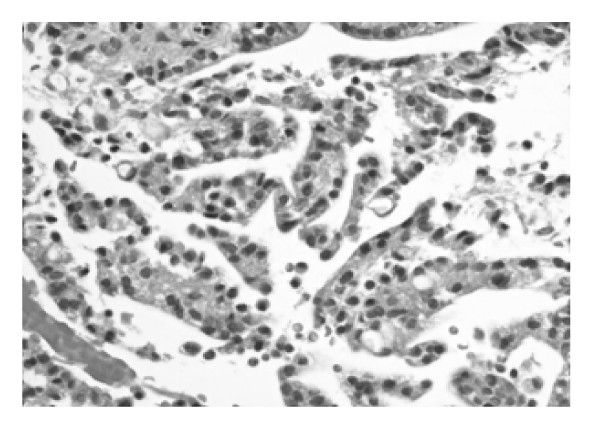
**MPE with areas of epithelioid morphology with occasional cytoplasmic clearing.** ×200; H&E.

**Figure 2:**
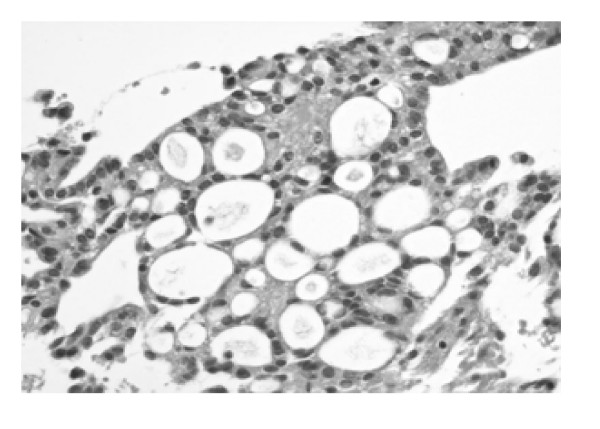
**MPE with areas showing a cribriform pattern of cells resembling a carcinoma.** ×400; H&E.

**Figure 3:**
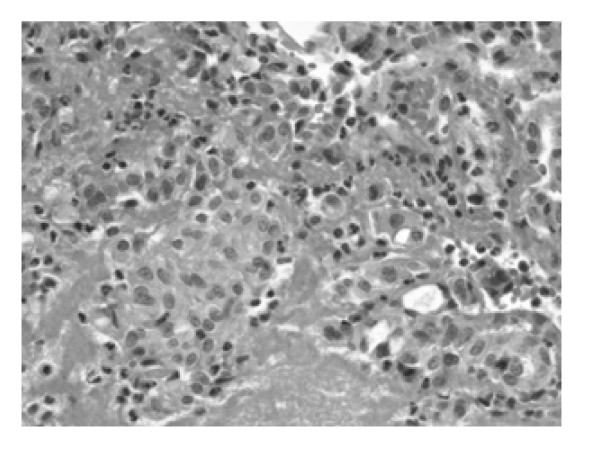
**MPE demonstrating solid sheets and cords of cells.** ×200; H&E.

Microscopic examination of the tissue obtained showed the classical morphological features of MPE with formation of pseudopapillae and pseudorosettes embedded in a myxoid stroma. The cells, which made up the pseudorosettes, had epithelioid morphology with occasional cells showing clear cytoplasm (figure 1). In addition, cribriform areas (figure 2), solid sheets and cords of cells resembling a carcinoma were also present (figure 3). The differential diagnosis was, MPE, metastatic carcinoma and chordoma.

**Figure 4:**
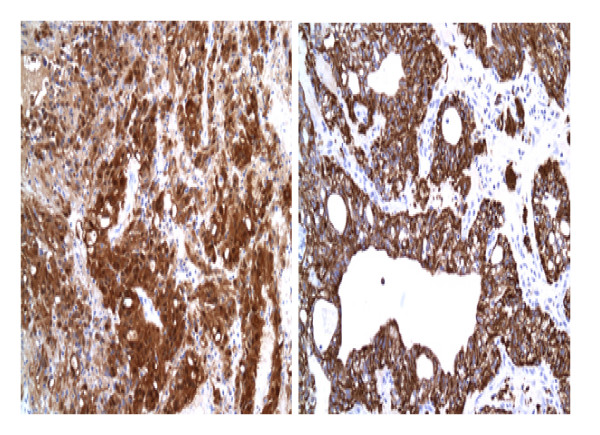
**A: Positive S-100 protein stain in the neoplastic cells indicating glial nature of the lesion.** ×200. 4B: GFAP positivity in the neoplastic cells. ×200.

**Figure 5:**
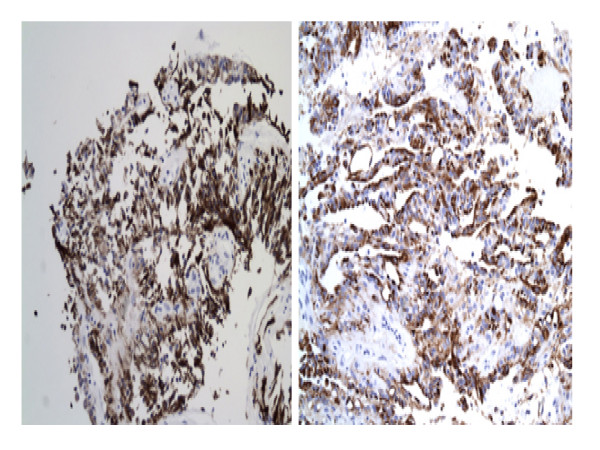
**Positive epithelial markers; AE1AE3 (A) and CAM5.**2 (B) in the neoplastic cells.

Immunohistochemically, the neoplastic cells showed strong, diffuse positive reaction with S-100 protein (figure 4a) and glial fibrillary acidic protein (GFAP) (figure 4b), indicating the glial nature of the lesion. The tumour cells in both cases showed strong positivity for cytokeratin markers, AE1AE3 (figure 5a), CAM 5.2 (figure 5b) and focally for cytokeratin 7. Both cases demonstrated a low proliferative index (< 2%) with Ki-67. The neoplastic cells were negative for cytokeratin 20, NSE, synaptophysin and neurofilament. Epithelial membrane antigen (EMA) focally stained luminal tips of occasional tumor cells. Histochemically, areas of mucoid degeneration were positive for alcian blue and periodic-acid-schiff (PAS) stains.

## Discussion

MPE is a glial tumour occurring almost exclusively in the region of the cauda equina and considered to be one of the most frequent primary tumours to occur in this location [3,11]. MPE was first described as a separate entity by Kernohan in 1931 [11] with isolated case reports subsequently appearing in the literature [[12]&[13]]. The clinical presentation depends on the location of the tumour. The majority of cauda equina and filum terminale tumours present with low back pain due to nerve root compression similar to our cases. Lower limb weakness and sphincter dysfunction are the two other common clinical manifestations. Intramedullary MPE arise from the ependymal lined cells of the filum terminale. The proposed histogenesis of extramedullary MPE in this location is the presence of ependymal rest of the neural tube during canalization and retrogressive differentiation [14,15] or from ectopic ependymal cell [16]. Recent study has suggested that radial glia is the cell of origin of ependymoma [17,18].

The classic morphology is easily recognizable, comprising multiple papillae covered by flattened to cuboidal cells embedded in a myxoid stroma and forming pseudorosettes. However, there are cases where the tumour obtains a solid growth pattern with aggregates of cells with "epithelial morphology" which in addition may show clearing of the cytoplasm. In such instances, metastatic carcinomas of renal origin and chordoma have to be ruled out by adequate clinical history and immunohistochemical stains.

It is well known that MPE stain positive with GFAP and S-100 protein. Cytokeratin positivity in MPE has been a subject of controversy with few cases reported showing positive cytokeratin expression in MPE [5,19,20]. Both our cases were positive for CAM5.2, AE1AE3, EMA and CK7 but negative for CK20.

Morphological variations in MPE can resemble metastatic carcinoma. An erroneous diagnosis may subject the patients to unnecessary metastatic work-up and additional adjuvant therapy. In addition, one should also consider the psychological implication of diagnosing a carcinoma. MPE has a better prognosis with tendency for late recurrence, except for some cases with aggressive behavior and seeding to the CNS [21]. MPE with clear cell changes and positive staining for S-100 protein, keratins and EMA can be misdiagnosed as chordoma, the latter being positive for Cytokeratin markers and S-100 protein but negative for GFAP. Although, metastatic carcinomas are positive for cytokeratins, these are consistently negative for GFAP.

MPE can be positive for Cytokeratins (CAM 5.2, AE1 AE3, and CK7) and focally for EMA. Given the similarities in morphology between metastatic carcinoma, chordoma and MPE, the diagnosis of MPE should be made in conjunction with clinical history including tumour location, immunohistochemical profile (co-expression of GFAP, S-100 protein and epithelial markers in MPE) and radiological findings. The recommended panel should include Cytokeratins (positive in MPE, metastatic carcinoma and chordoma), GFAP (positive in MPE and negative in metastatic carcinoma and chordoma), and S-100 protein (positive in MPE, chordoma but negative in most metastatic carcinomas).

The majority of MPE are slow growing gliomas with a tendency for local recurrence. Prognosis depends on complete surgical resection of the tumor. The risk of metastasis is very low and rare. The overall prognosis of MPE is much better than metastatic carcinoma and the latter has to be ruled out as they can have similar morphological features.

In conclusion, we reemphasize the importance of using a panel of immunohistochemical stains to differentiate between MPE and other tumour entities to avoid misdiagnosis.

## Competing interests

The authors declare that they have no competing interests.

## Authors' contributions

Both authors had contributed equally to the preparation of the manuscript. Both authors read and approved the final manuscript.
